# Job Satisfaction Goes a Long Way: The Mediating Role of Teaching Satisfaction in the Relationship between Role Stress and Indices of Psychological Well-Being in the Time of COVID-19

**DOI:** 10.3390/ijerph192417071

**Published:** 2022-12-19

**Authors:** Anita Padmanabhanunni, Tyrone Brian Pretorius

**Affiliations:** Department of Psychology, University of the Western Cape, Bellville 7530, South Africa

**Keywords:** anxiety, COVID-19, depression, role conflict, role ambiguity, teachers

## Abstract

The COVID-19 pandemic and its containment measures has resulted in drastic changes in the educational landscape. Teachers had to rapidly adapt to shifts in their work-related roles and responsibilities. This situation likely led to role stress and affected the levels of job satisfaction, mental health and general life satisfaction. In this study, we examined the role of teachers’ job satisfaction in the relationship between role stress and indices of psychological well-being. The participants were South African school teachers (N = 355) who completed the Role Orientation Questionnaire, the Teaching Satisfaction Scale, the Center for Epidemiological Studies Depression Scale, the trait scale of the State-Trait Anxiety Inventory and the Satisfaction with Life Scale. In addition to descriptive statistics and correlations, path analysis was performed to determine the mediating role of teaching satisfaction. Increased levels of teaching satisfaction were associated with decreased levels of depression and anxiety and increased levels of life satisfaction. Teaching satisfaction also mediated the relationship between role conflict, as well as role ambiguity and anxiety. The results indicated that teaching satisfaction is a critical protective factor for teachers. Thus, improving task significance and enhancing the meaning associated with the teaching profession may help promote the adaptive functioning of teachers.

## 1. Introduction

As part of the COVID-19 containment measures, several countries enforced total or partial closures of their educational institutions. Because conventional classroom teaching was no longer possible, schools had to rapidly migrate to remote online learning and teaching systems [1]. Teachers were also forced to adapt to this new system, with many of them not having the necessary competencies or technological experience to navigate the online delivery of education. Several scholars [1,2] have indicated that remote online education in the context of the pandemic substantially differs from deliberate and well-designed online teaching. Under standard conditions, developing an online course usually takes 6–9 months. However, in the context of COVID-19, teachers had to transition within days or weeks [1]. They also had to help their students navigate the online environment, identify new methods to motivate their students, and mitigate the effect of the pandemic on their students and their parents and the educational system as a whole [3]. Furthermore, teachers had to contend with the implications of the pandemic in their personal lives and relationships. In this context, several factors have been identified as having an impact on the mental health and well-being of teachers, such as increased workloads and increased domestic responsibilities, including child care, home schooling and caring for significant others [4]. 

An early meta-analysis [2] focusing on the prevalence of mental health problems among teachers reported increased levels of depression, anxiety, burnout and insomnia among this population. These levels of psychological distress were higher than those reported in a meta-analysis of studies among the general population [5], suggesting that the pandemic had distinctive effects on the mental health of teachers. Studies on teachers from different countries have further highlighted their deteriorating mental health and well-being (e.g., Australia [3], Spain [2] and China [6]). For example, a longitudinal study [7] in the United Kingdom reported an increase in the levels of anxiety among teachers following school closures and transition to remote learning. Similarly, studies in the United States have reported increased levels of anxiety [8] and depression [9] among teachers who had to transition to virtual teaching modes. These changes in the job demands of teachers resulted in higher levels of burnout, a condition that was already prevalent among teachers before the pandemic [10]. 

This study was performed in South Africa, where substantial digital inequalities and disparities in access to resources meant that remote online learning and teaching were not sustainable [4,11]. In March 2020, schools were initially closed, as part of the COVID-19 containment measures. However, by February 2021, during the third wave of the pandemic, teachers were expected to transition back to conventional schooling [11]. In addition to ensuring the resumption of teaching and learning activities, teachers were responsible for implementing COVID-19 safety protocols in the school environment, such as social distancing, mask wearing and sanitization. Generally, in South Africa, several schools are located in disadvantaged communities or in rural areas that lack an adequate infrastructure, resulting in overcrowding in classrooms and inadequate resources (e.g., limited access to personal protective equipment and clean running water), to implement the aforementioned safety measures [12]. This situation may increase the levels of stress and anxiety among teachers toward their ability to protect themselves and their significant others from infection [13]. It may also affect their levels of job satisfaction or teaching satisfaction, which is defined as a sense of contentment with one’s job, and serves as a motivational factor [4]. Some empirical studies [14,15] have highlighted the associations between job satisfaction and various other work-related experiences, including career commitment, happiness, job performance, intention to quit and burnout. 

With COVID-19, dramatic shifts have been observed in the job characteristics, roles and responsibilities associated with the teaching profession, which in turn affected the levels of job satisfaction and psychological well-being of teachers [1,11]. These abrupt shifts in education delivery and teacher workloads represented a major source of role stress [16]. According to role theory [17] role conflict (RC) and role ambiguity (RA) are two features of the experience of role stress. RC represents one aspect of role stress, and occurs in the presence of various and often diverging role expectations imposed on an individual [17,18]. RC also occurs in the presence of a discrepancy between the expected roles of an individual and their personal values. These conflicting expectations may lead to psychological tension, uncertainty and increased stress, because the employee cannot simultaneously fulfil every expected role. RA, a second feature of role stress, occurs when the information available to effectively perform work duties is insufficient or inadequate [17]. This causes employees to be uncertain regarding their work-related objectives, roles and responsibilities. The expectations of managers and colleagues may also be unclear. Although most people experience role stress negatively, it has the potential to promote creativity, flexibility and adaptation to changing circumstances [17]. 

This study is framed within the job characteristics model (JCM) [19], which proposes that job satisfaction (i.e., a positive emotional state arising from the individual’s perception that their job achieves or facilitates their job values) is affected by five job characteristics: skill variety, task identity, task significance, autonomy and feedback. These job characteristics affect job satisfaction through three psychological states: (1) the extent to which a job is experienced as meaningful to self and others, (2) the degree to which an individual experience a sense of personal responsibility for the outcomes of the job and (3) the extent to which an individual knows how successful their work has been. Several studies have supported the JCM model [20,21], and various meta-analyses have confirmed the validity of the association between job characteristics and psychological outcomes [22]. In the context of teachers, the pandemic considerably affected the characteristics of their work, which may have also affected their job satisfaction levels. Therefore, in this study, we examined the potential role of teachers’ job satisfaction in the relationship between role stress and indices of psychological well-being. 

Several studies performed before and during the pandemic on different population groups (e.g., hotel-industry workers [23], professional women in China [24], and social workers [25]) have confirmed that job satisfaction mediates the relationship between role stress and mental health outcomes. In our study, we used both positive (i.e., life satisfaction) and negative (i.e., depression and anxiety) indicators of well-being. With respect to the mediating role of teaching satisfaction, we thus hypothesized that:

**Hypothesis** **1.** *Teaching satisfaction mediates the relationship between RA and depression.*

**Hypothesis** **2.** *Teaching satisfaction mediates the relationship between RA and depression.*

**Hypothesis** **3.** *Teaching satisfaction mediates the relationship between RA and life satisfaction.*

**Hypothesis** **4.** *Teaching satisfaction mediates the relationship between RC and depression.*

**Hypothesis** **5.** *Teaching satisfaction mediates the relationship between RC and anxiety.*

**Hypothesis** **6.** *Teaching satisfaction mediates the relationship between RC and life satisfaction.*

## 2. Materials and Methods

### 2.1. Participants

School teachers (N = 355) were recruited from all provinces of South Africa, with the majority based in the Western Cape (82.3%). Most of the sample were women (76.6%), who were teachers working at primary schools (grades 1–7, 61.1%) and were based in urban areas (61.7%). The mean age of the participants was 41.9 (SD = 12.42). With regard to their personal experiences of COVID-19, 23.4% reported having been infected, 92.1% reported knowing people who had been infected, 63.9% reported losing a family member and 27% reported losing a colleague. We also compared our sample to population data reported by an international survey undertaken in South Africa [26], and found no significant differences between our sample and the population in terms of gender (χ^2^ = 0.06, *p* > 0.05 and age (t = 1.68, *p* > 0.05). 

### 2.2. Instruments

All participants completed a brief demographic survey and the following instruments: the Role Orientation Questionnaire (ROQ) [27], the Teaching Satisfaction Scale (TSS) [28], the Center for Epidemiological Studies Depression Scale (CES-D) [29], the trait scale of the State-Trait Anxiety Inventory (STAI-T) [30], and the Satisfaction with Life Scale (SWLS) [31]. 

The ROQ consists of two subscales, RC and RA, which assess the perceptions of work roles. The RA dimension contains eight items, and measures the extent to which work roles are perceived as being ambiguous, with unclear expectations. The RC dimension contains six items, and measures the extent to which employees experience incompatible or conflicting demands [32]. Responses to the scale items are scored on a 6-point scale ranging from 1 (definitely not true of my job) to 6 (definitely true of my job). In the original study, Rizzo and colleagues reported satisfactory reliability coefficients (α = 0.82 and α = 0.87 for RC and RA, respectively) and indicated that the relationship between these two constructs and several organizational (e.g., leadership) and personal (e.g., job satisfaction and anxiety) variables, served as evidence of validity [27]. 

The TSS is a measure of the job satisfaction experienced by teachers. It consists of five items scored on a 5-point Likert scale ranging from 1 (strongly disagree) to 5 (strongly agree). In the original study, Ho and Au reported a satisfactory reliability estimate (α = 0.77) and provided evidence of instrument validity [28]. Other recent studies have also reported satisfactory estimates of internal consistency. For example, Nalipay et al. reported an α value of 0.85 [33], whereas Han et al. reported an α value of 0.92 [34].

The CES-D is one of the most widely used measures of depression. It consists of 20 items scored on a 4-point scale, ranging from 0 (rarely or none of the time) to 3 (most or all of the time). In the original study, Radloff reported reliability estimates ranging between 0.84 and 0.90 across different samples and provided evidence of construct validity for the scale [29]. In addition, a review of the assessment of depression in older adults indicated that the internal consistency of the CES-D in a number of studies was highly satisfactory (α = 0.81–0.93) [35]. The CES-D was also reported to be a reliable measure of depression among student samples from South Africa (e.g., α = 0.90 [36] and α = 0.92 [37]).

The STAI-T is a measure of trait anxiety. It consists of 20 items scored on a 4-point scale ranging from 1 (almost never) to 4 (almost always). According to Spielberger, the STAI-T has a median α coefficient of 0.90 and test–retest coefficients ranging between 0.73 and 0.86 for male and female high-school and college students [30]. In addition, a reliability generalization study reviewing 816 articles on the STAI reported an average internal consistency coefficient of 0.91 and an average test–retest coefficient of 0.88 across these articles [38]. More contemporary studies have reported similar satisfactory reliability for the STAI-T (e.g., 0.84–0.88 [39] and 0.91–0.93 [40]). Padmanabhanunni and Pretorius reported an internal consistency estimate of 0.84 for a student sample from South Africa [41].

The SWLS is a five-item scale that measures the cognitive component of subjective well-being. Responses to these five items are scored on a 7-point scale, with scale anchors ranging from 1 (strongly disagree) to 7 (strongly agree). According to Diener and colleagues, the SWLS has a high internal consistency estimate (α = 0.87), and the correlations between the SWLS and other measures of well-being provide evidence of concurrent validity [31]. Similarly, more contemporary studies have reported highly satisfactory estimates of reliability (e.g., α = 0.86 [42] and α = 0.84 [43]). The SWLS has also been used in a previous study with a student sample from South Africa, and a Cronbach α of 0.89 was reported [44]. In addition, Pretorius and Padmanabhanunni confirmed the reliability, validity and unidimensionality of the SWLS among a sample of school teachers, by using both classical test theory and item-response theory [45].

### 2.3. Procedure

In this cross-sectional study, we used Google Forms to construct an electronic survey. The administrators of various teacher groups gave permission for the link to be posted on their websites. In addition, the school liaison unit at the university distributed the link to various schools where they had contacts. Data were collected in April–July 2021, which coincided with the third wave of the pandemic, which was associated with a considerable increase in the number of cases because of the Delta variant [46]. 

### 2.4. Ethics

This study was performed in accordance with the Declaration of Helsinki. Ethical approval was obtained from the Humanities and Social Sciences Ethics committee of the University of the Western Cape (reference number: HS21/3/8). All participants were required to provide informed consent on the landing page of the link before being allowed to proceed. They were also assured on the landing page that participation was entirely voluntary and that they could withdraw at any time. 

### 2.5. Data Analysis

**Figure 1:**
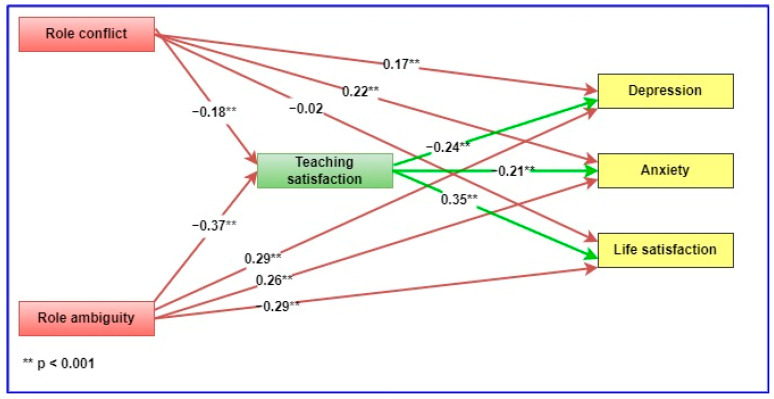
Path-analysis model of the relationship between role stress, teaching satisfaction and psychological well-being.

Descriptive statistics (means and standard deviations), reliabilities (α and ω) and intercorrelations (Pearson’s correlation) between study variables were obtained using IBM SPSS Statistics version 28 for Windows (IBM Corp., Armonk, NY, USA). Because of some concerns regarding coefficient α underestimating the true reliability in multi-item measurement scales, we reported on both the α and ω coefficients [47,48]. An omega analysis was performed with the OMEGA macro, written by Hayes and Coutts for SPSS [48]. To examine the mediating effects of teaching satisfaction, path analysis was performed using IBM Amos version 28 for Windows. Maximum likelihood estimation was used with bootstrapped 95% confidence intervals (CIs). In the mediation model, RC and RA were specified as predictors, the indices of psychological well-being were specified as outcome variables, and teaching satisfaction was specified as the presumed mediator (see Figure 1). A significant indirect effect of RC and RA through teaching satisfaction on the indices of psychological well-being was regarded as indicative of a mediating effect.

## 3. Results

All descriptive statistics, intercorrelations and reliabilities of the study variables are reported in Table 1. All scales demonstrated satisfactory reliability (α and ω = 0.83–0.93). RC was positively related to the negative indices of psychological well-being (anxiety: r = 0.27, *p* < 0.001, 95% CI = [0.17, 0.36; depression: r = 0.23, *p* < 0.001, 95% CI = [0.12, 0.32]) but not significantly related to life satisfaction (r = −0.09, *p* = 0.09, 95% CI = [−0.19, 0.01]). However, RA was positively related to both positive and negative indicators of psychological well-being (anxiety: r = 0.34, *p* < 0.001, 95% CI = [0.25, 0.43]; depression: r = 0.38, *p* < 0.001, 95% CI = [0.29, 0.47]; life satisfaction: r = −0.42, *p* < 0.001, 95% CI = [−0.50, −0.33]). These results indicate that higher levels of RC and RA are associated with higher levels of depression and anxiety.

As further shown in Table 1, teaching satisfaction was negatively related to the predictors of psychological well-being (RC: *r* = −0.19, *p* < 0.001, 95% CI = [−0.29, −0.09]; RA: *r* = −0.38, *p* < 0.001, 95% CI = [−0.46, −0.28]) and the negative indices of psychological well-being (anxiety: *r* = −0.35, *p* < 0.001, 95% CI = [−0.44, −0.25]; depression: *r* = −0.38, *p* < 0.001, 95% CI = [−0.47, −0.29]). Teaching satisfaction was also positively related to life satisfaction (*r* = 0.46, *p* < 0.001, 95% CI = [0.37, 0.54]). These results indicate that higher levels of role stress (conflict and ambiguity) are associated with lower levels of teaching satisfaction. They also indicate that higher levels of teaching satisfaction are associated with lower levels of depression and anxiety and higher levels of life satisfaction.

Figure 1 shows the path-analysis model used to test the mediating role of teaching satisfaction, together with the standardized estimates resulting from the analysis. The figure also shows the indirect effects of RC and RA on the indices of psychological well-being.

The direct and indirect effects resulting from the path-analysis model in Figure 1 are presented in Table 2. With the exception of the association between RC and life satisfaction, the results in Table 2 confirm that all of the direct and indirect effects were significant.

The results in Table 2 support all the hypotheses. More specifically, the results indicate the following:

- RA was positively associated with depression (β = 0.26, *p* < 0.001, 95% CI [0.20, 0.36]) and anxiety (β = 0.29, *p* < 0.001, 95% CI [0.17, 0.33]) and negatively associated with life satisfaction (β = −0.29, *p* < 0.001, 95% CI [−0.36, −0.20]).
- RC was positively associated with depression (β = 0.17, *p* < 0.001, 95% CI [0.09, 0.24]) and anxiety (β = 0.22, *p* < 0.001, 95% CI [0.13, 0.30]).
- Teaching satisfaction was negatively associated with depression (β = −0.24, *p* < 0.001, 95% CI [−0.33, −0.16]) and anxiety (β = −0.21, *p* < 0.001, 95% CI [−0.30, −0.11]) and positively associated with life satisfaction (β = 0.35, *p* < 0.001, 95% CI [0.26, 0.44]).
- Teaching satisfaction mediated the relationship between RC on the one hand and anxiety (β = 0.04, *p* < 0.001, 95% CI [0.02, 0.07]), depression (β = 0.04, *p* < 0.001, 95% CI [0.02, 0.07]) and life satisfaction (β = −0.06, *p* < 0.001, 95% CI [−0.10, −0.03]) on the other hand.
- Teaching satisfaction mediated the relationship between RA on the one hand and anxiety (β = 0.08, *p* < 0.001, 95% CI [0.05, 0.12]), depression (β = 0.09, *p* < 0.001, 95% CI [0.06, 0.13]) and life satisfaction (β = −0.13, *p* < 0.001, 95% CI [−0.18, −0.09]) on the other hand.

## 4. Discussion

With COVID-19 radically changing the educational landscape, several changes in the roles and responsibilities associated with being a teacher were observed. In this study, we investigated the role of teacher job-satisfaction in the relationship between role stress and three common indices of psychological well-being, namely, depression, anxiety and life satisfaction. In the following, we highlight our key findings. 

Firstly, high levels of role stress were associated with adverse psychological outcomes in the form of increased depression and anxiety. This finding lends further support to studies, for example [2], focusing on the mental health of teachers during the pandemic. For example, a study of school principals [49] reported increased levels of depression among those who were expected to assume a range of tasks, including teacher supervision, administration, teaching, school management and student discipline. In a similar vein to those of school principals, the roles of teachers during the pandemic have changed, to include a range of unanticipated tasks, such as implementing COVID-19 safety-protocols in classrooms (e.g., sanitizing classrooms and maintaining social distancing), monitoring adherence among students to these measures and guiding and motivating students and their parents in terms of online learning and the return to conventional schooling [13]. These diverse roles and responsibilities likely increased the levels of psychological distress and contributed to depression and anxiety. In addition, the uncertainty associated with face-to-face teaching and the increased perception of vulnerability to infection while commuting to work and while interacting in person with students and staff, may have contributed to increased levels of anxiety [50]. 

Given that a large percentage of South African teachers (approximately 46%) have comorbid physical health-conditions, worrying about the implications of exposure to the virus in the classroom context may have increased their levels of anxiety [50]. Within the framework of the JCM, changes in the tasks expected of teachers and challenges in meeting the expectations of others may affect their sense of fulfilment within their profession. In addition, difficulty adhering to COVID-19 safety protocols because of poor school infrastructure or limited access to personal protective equipment may generate feelings of hopelessness among teachers, and contribute to depression [50]. Moreover, being unable to manage online-learning scenarios and increased workloads may cause teachers to negatively appraise their work (e.g., indicating that they have failed as teachers), which may in turn precipitate feelings of depression [51]. 

Secondly, in line with the JCM, higher levels of role stress were associated with lower levels of life satisfaction and teaching satisfaction. Since the onset of the pandemic, the roles of teachers have become more complex. They had to redesign courses for remote online-teaching, guide students through the online learning environment and manage the personal impact of the pandemic on their own lives [1]. They also had to meet the shifting expectations of the educational sector, including the return to conventional teaching. In several schools in South Africa, particularly those in rural areas, teachers were burdened with increased teaching and managerial responsibilities, because of the lack of resources [13]. Under these circumstances, role stress may have contributed to emotional exhaustion and burnout and thereby reduced the levels of life satisfaction and teaching satisfaction. Although we did not assess these variables, robust evidence [51] confirms the association between role stress and reduced job satisfaction and psychological well-being. 

According to the theory of symbolic interactionism [52], social interactions play a central role in people’s identity construction and personal values. Increased role stress is likely to affect teaching identity and cause teachers to re-evaluate their personal values and the extent to which their work roles are satisfactory. Negative evaluations may lead to decreased levels of teaching satisfaction. In addition, increased work-related responsibilities with no commensurate increase in income may lead to feelings of under-compensation among teachers, and result in decreased levels of teaching satisfaction. Finally, increased workloads are typically associated with reduced time for leisure activities and rest periods, and this may adversely affect the levels of life satisfaction [51]. 

Thirdly, higher levels of teaching satisfaction were associated with lower levels of depression and anxiety and higher levels of life satisfaction. Teaching satisfaction mediated the relationship between RC as well as RA and anxiety. These findings conform with those of previous studies, for example [53], which linked job satisfaction with a host of positive variables, including a reduced risk of burnout and improved physical and psychological well-being. According to the JCM [19], people experience a sense of job satisfaction when they perceive their work as meaningful. In keeping with this perspective, teachers who appraise their work as contributing to the greater good of their students and communities may experience an increased sense of teaching satisfaction. The meaning that they derive from work translates into greater life satisfaction, and safeguards them against adverse mental-health outcomes [53]. 

The results of the present study have practical implications. For example, the results suggest that promoting teaching satisfaction may be a protective resource for mental health and well-being among this population. One way to achieve this is by enhancing task significance, that is, the extent to which teachers appraise their work as improving the lives of others [53]. Work is generally perceived as meaningful when it has a positive and prosocial effect on others [53]. Task significance can be promoted by enhancing the autonomy of teachers and their sense of shared decision-making. Evidence-based interventions, such as acceptance and commitment therapy, can also enhance meaning by encouraging people to identify their personal values and to engage in activities that align with their beliefs [54]. For teachers, this may involve the exploration of their original reasons for entering the profession, highlighting positive experiences at work and the considerable effect that they have on their students and communities. Acceptance and commitment therapy is effective when delivered in person or through a web-based format, and it is associated with favorable outcomes, including reducing burnout, depression and anxiety [54]. In South Africa, mentoring has been identified as a potential resource in supporting teachers [55]. Through coaching and the provision of psycho-social support, mentoring can assist with role conflict and role ambiguity through the development of heightened self-confidence, in solving job-related challenges and increasing job efficiency [56]. Mentoring is also cost-effective, and teachers may be more willing to discuss their challenges with a senior colleague working within the same context and who is experiencing similar challenges. 

This study has certain limitations. First, its cross-sectional design precluded causal inferences. Therefore, longitudinal research is required to identify changes in teaching satisfaction and their association with mental-health outcomes and life satisfaction. Second, all data were collected online, meaning that the sample comprised only a group of teachers who had access to electronic resources and were interested in the survey. In addition, most of the respondents were from one geographical area, and were largely women. Hence, our results should be tested and replicated by using data collected with different methods and reflecting a diverse sample of teachers. 

## 5. Conclusions

Teacher job-satisfaction has significant and far-reaching implications, including higher instructional quality and provision of greater support to students. Teachers who experience a sense of job satisfaction are less susceptible to stress sand burnout, and more likely to remain in the profession. It therefore remains vital to investigate teaching satisfaction in the context of the COVID-19 pandemic, given the impact of disease containment measures on the education sector. This is the first study, to the authors’ knowledge, to investigate the role of teaching satisfaction in the relationship between role stress and psychological well-being in the context of COVID-19. Our findings generally underscore the significance of teacher satisfaction for mental health and life satisfaction. This suggests that teaching satisfaction may serve as a critical protective factor against adverse psychological outcomes. Improving task significance and enhancing the meaning associated with the teaching profession may help promote the adaptive functioning of teachers in the context of the pandemic. Interventions such as ACT and implementing formal mentoring support for teachers represent important avenues for enhancing job satisfaction. In addition, educational policies aimed at capacity building and the promotion of school climates conducive to collaboration, support and mutual goal-setting can be instrumental in teacher satisfaction. This study underscores the importance of attending to the occupational and personal wellbeing of teachers in the context of a societal crisis. 

## Figures and Tables

**Table 1:** Descriptive Statistics, Intercorrelations between, and Reliabilities of, Study Variables.

Variable and Indices	1	2	3	4	5	6
1. Role conflict	—					
2. Role ambiguity	0.04	—				
3. Depression	0.23 ***	0.38 ***	—			
4. Anxiety	0.27 ***	0.34 ***	0.74 ***	—		
5. Life satisfaction	−0.09	−0.42 ***	−0.55 ***	−0.52 ***	—	
6. Teaching satisfaction	−0.19 ***	−0.38 ***	−0.38 ***	−0.35 ***	0.46 ***	—
Mean	30.4	14.7	22.0	44.9	21.9	17.3
SD	8.2	5.7	12.2	10.3	7.3	4.7
Alpha	0.83	0.83	0.92	0.91	0.90	0.87
Omega	0.83	0.83	0.93	0.91	0.90	0.87

*** *p* < 0.001.

**Table 2:** Direct and Indirect Effects of Role Stress on indices of Psychological Well-Being.

Effect	Beta	SE	β	95% CI	*p*
Direct Effects					
Role conflict → anxiety	0.27	0.06	0.22	[0.13, 0.30]	0.001
Role conflict → depression	0.25	0.07	0.17	[0.09, 0.24]	0.001
Role conflict → life satisfaction	−0.01	0.04	−0.02	[−0.09, 0.07]	0.792
Role ambiguity → anxiety	0.47	0.09	0.26	[0.17, 0.33]	0.001
Role ambiguity → depression	0.62	0.10	0.29	[0.20, 0.36]	0.001
Role ambiguity → life satisfaction	−0.37	0.06	−0.29	[−0.36, −0.20]	0.001
Role conflict → teaching satisfaction	−0.10	0.03	−0.18	[−0.26, −0.09]	0.001
Role ambiguity → teaching satisfaction	−0.31	0.05	−0.37	[−0.46, −0.27]	0.001
Teaching satisfaction → anxiety	−0.47	0.13	−0.21	[−0.30, −0.11]	0.001
Teaching satisfaction → depression	−0.64	0.14	−0.24	[−0.33, −0.16]	0.001
Teaching satisfaction → life satisfaction	0.54	0.09	0.35	[0.26, 0.44]	0.001
Indirect Effects					
Role conflict → teaching satisfaction → anxiety	0.05	0.02	0.04	[0.02, 0.07]	0.001
Role conflict → teaching satisfaction → depression	0.06	0.02	0.04	[0.02, 0.07]	0.001
Role conflict → teaching satisfaction → life satisfaction	−0.05	0.02	−0.06	[−0.10, −0.03]	0.001
Role ambiguity → teaching satisfaction → anxiety	0.14	0.04	0.08	[0.05, 0.12]	0.001
Role ambiguity → teaching satisfaction → depression	0.19	0.05	0.09	[0.06, 0.13]	0.001
Role ambiguity → teaching satisfaction → life satisfaction	−0.17	0.04	−0.13	[−0.18, −0.09]	0.001

## Data Availability

The raw data supporting the conclusions of this article will be made available by the authors, without undue reservation.
